# Supplementary material for: Neutrophil metabolomics in severe COVID-19 reveal GAPDH as a suppressor of neutrophil extracellular trap formation
Source: Nat Commun. 2023 May 5;14:2610. doi: 10.1038/s41467-023-37567-w (PMC10162006; doi:10.1038/s41467-023-37567-w)
Supplement: Supplementary file 1 — Supplementary Information [file 41467_2023_37567_MOESM1_ESM.pdf]

Neutrophil metabolomics in severe COVID-19 reveal GAPDH as a suppressor of neutrophil extracellular trap formation

Yafeng Li[1], Jessica S Hook[2], Qing Ding[1], Xue Xiao[2,3], Stephen S Chung[4], Marcel Mettlen[5], Lin Xu[2,3], Jessica G Moreland[2,6], Michalis Agathocleous[1,2*]

[1]Children's Medical Center Research Institute, University of Texas Southwestern Medical Center, Dallas, TX, USA

[2]Department of Pediatrics, University of Texas Southwestern Medical Center, Dallas, TX, USA

[3]Quantitative Biomedical Research Center, Department of Population and Data Sciences, University of Texas Southwestern Medical Center, Dallas, TX, USA

[4]Department of Internal Medicine, Division of Hematology and Oncology, University of Texas Southwestern Medical Center, Dallas, TX, USA

[5]Department of Cell Biology, Quantitative Light Microscopy Core, University of Texas Southwestern Medical Center, Dallas, TX, USA

[6]Department of Microbiology, University of Texas Southwestern Medical Center, Dallas, TX, USA.

*Correspondence to: michail.agathokleous@utsouthwestern.edu

FIGURE S1

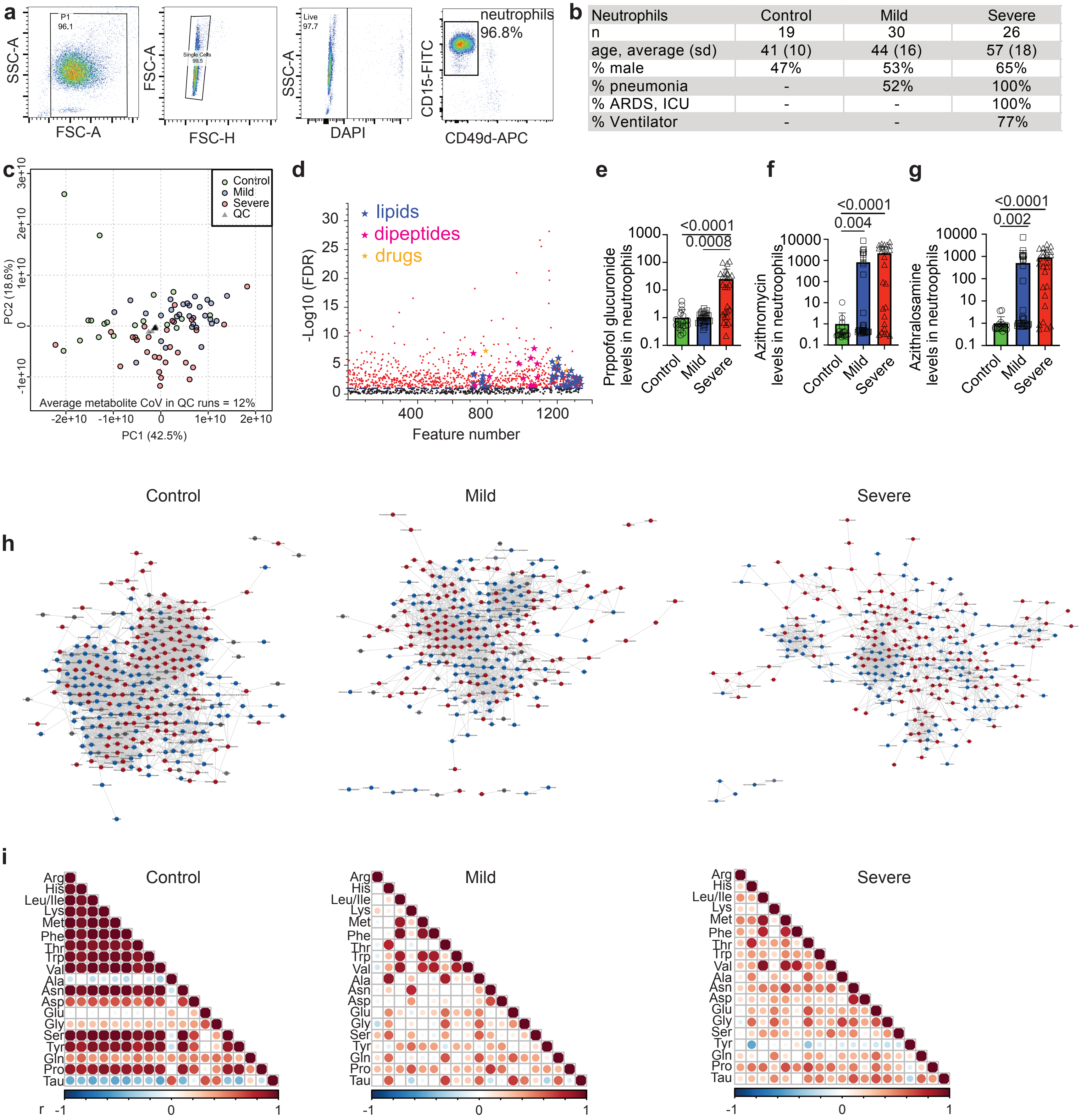

Neutrophils	Control	Mild	Severe
n	19	30	26
age, average (sd)	41 (10)	44 (16)	57 (18)
% male	47%	53%	65%
% pneumonia	-	52%	100%
% ARDS, ICU	-	-	100%
% Ventilator	-	-	77%

Supplementary Figure 1. Analysis of the neutrophil metabolome in COVID-19 patients and healthy controls
(a) Flow cytometry gating of neutrophils in polymorphonuclear leukocyte samples.
(b) Patient characteristics in the neutrophil metabolomics cohort
(c) PCA plot of raw data including quality control (QC) samples
(d) Untargeted metabolomics analysis showing features that significantly differ between healthy controls, mild or severe COVID-19 patients.
(e-g) Levels of propofol or azithromycin metabolites in neutrophils of the indicated groups.
(h) Correlation analysis shown in Fig. 1i-k with all metabolites labeled.
(i) Correlation of the levels of amino acids in healthy controls, mild COVID-19 or severe COVID-19 patient neutrophils.
Data represent mean ± st.dev. p values are shown on the graphs. Statistical significance was assessed with Kruskal-Wallis test (e-g). n=19 for healthy controls, n=30 for mild and n=26 for severe COVID-19 patients.

FIGURE S2

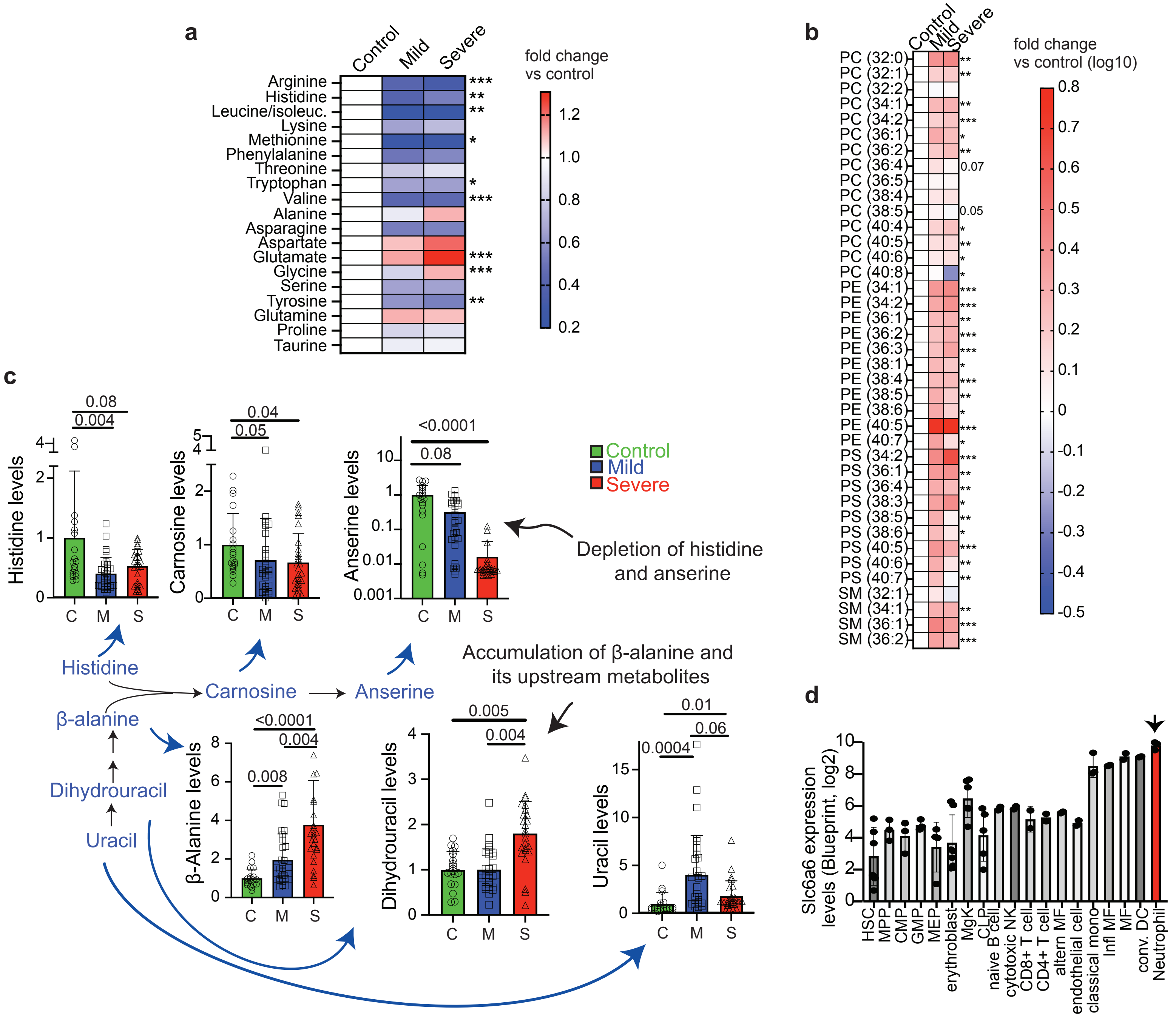

Supplementary Figure 2: Changes in amino acid and lipid metabolite levels in COVID-19 patient neutrophils. (a-b) Heatmaps showing changes in amino acid and lipid levels in neutrophils of COVID-19 patients. PC denotes phosphatidylcholines, PE phosphatidylethanolamines, PS phosphatidylserines and SM sphingomyelins.
(c) Changes in levels of metabolites in the histidine/β-alanine pathway: Depletion of histidine and its dipeptides and accumulation of uracil degradation metabolites and β-alanine in patients. The Y-axis represents signal intensity for these metabolites normalized to controls.
(d) Expression of Slc6a6 in neutrophils and other human hematopoietic and immune cell types (data from BLUEPRINT database, n=2-7 samples per cell type).
Data represent mean ± st.dev. Statistical significance was assessed with one-way ANOVA (a,b) or two tailed t test (c) followed by multiple comparisons correction using the full metabolomics data with 287 metabolites. In a and b, *p < 0.05, **p < 0.01, ***p < 0.001, and exact p values are provided in Supplementary Datasets.
Samples were from healthy controls (n=19), mild (n=30) or severe (n=26) COVID-19 patients.

FIGURE S3

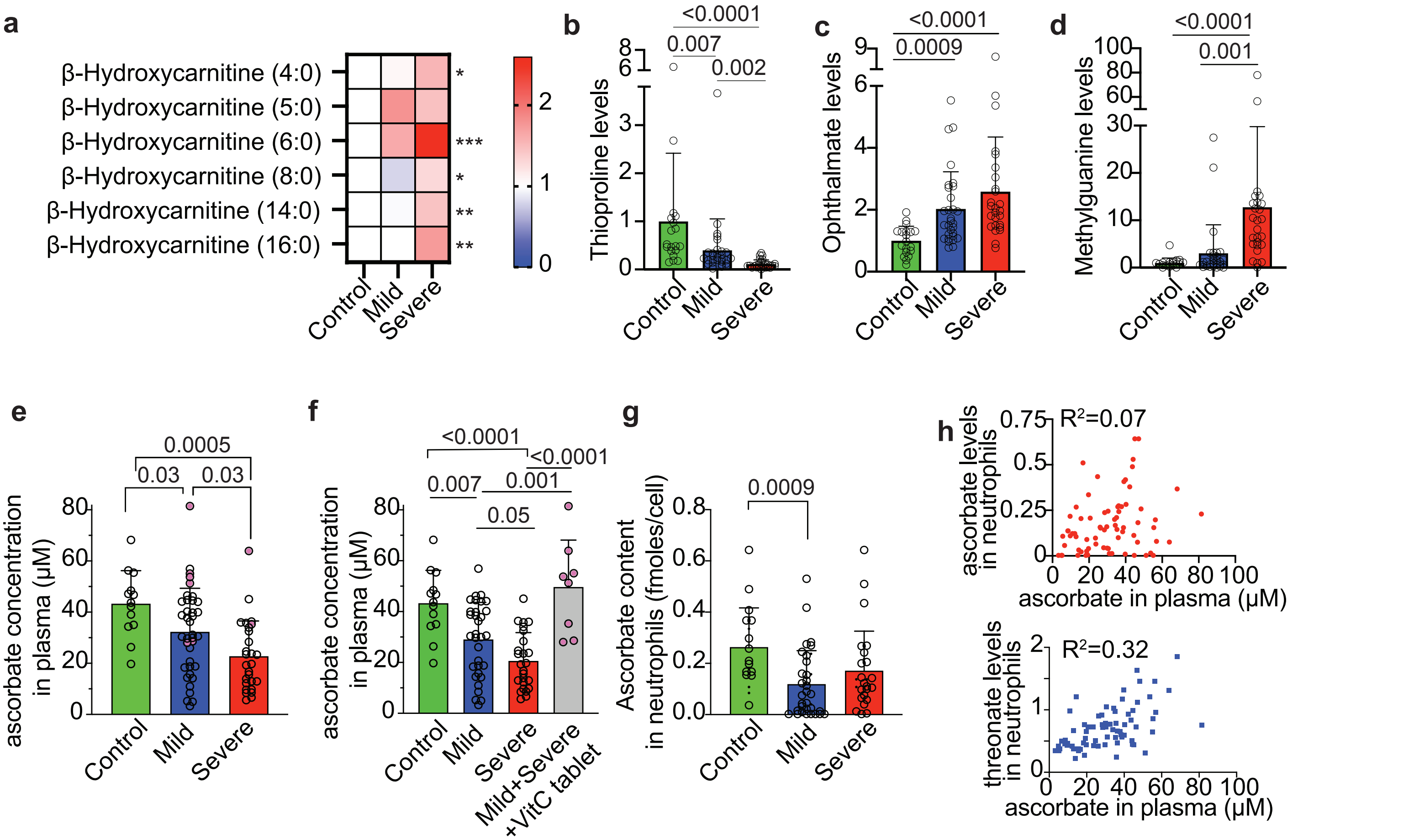

Supplementary Figure 3: Changes in levels of redox and antioxidant metabolites including ascorbate in COVID-19.
(a-d) Changes in levels of redox-related metabolites in neutrophils from COVID-19 patients and controls. Samples were from healthy controls (n=19), mild (n=30) or severe (n=26) COVID-19 patients.
(e-g) Ascorbate levels in plasma or neutrophils. In (e), samples from patients who received a vitamin C tablet are marked purple and these are shown in a separate column in (f). Vitamin C tablet in (f) contained 250–1000 mg of ascorbic acid.
In e, the number of samples in each group is: Control n = 13, Mild n = 35, Severe n = 26. In g, the number of samples in each group is: Control n = 19, Mild n = 30, Severe n = 26.
(h) Correlation of plasma ascorbate with neutrophil threonate or ascorbate levels in COVID-19 patients and controls. Plot shows samples for which both neutrophil and plasma measurements were available (n = 69).
Data represent mean ± st.dev. Statistical significance was assessed with one-way ANOVA (a) or two tailed t test (b-d) followed by multiple comparisons correction using the full metabolomics data with 287 metabolites. Or one-way ANOVA (e, f), or Kruskal-Wallis test (g), followed by multiple comparisons correction. p values are labeled in b-g on the corresponding graphs.

FIGURE S4

a

96 well plate ⟶ spinning disk confocal microscopy

6-9 hour time-lapse imaging
frame interval 2-4 minutes
~40 movies in parallel
Hoechst 33342:cell-permeable DNA stain
Sytox Green:cell-impermeable DNA stain

b

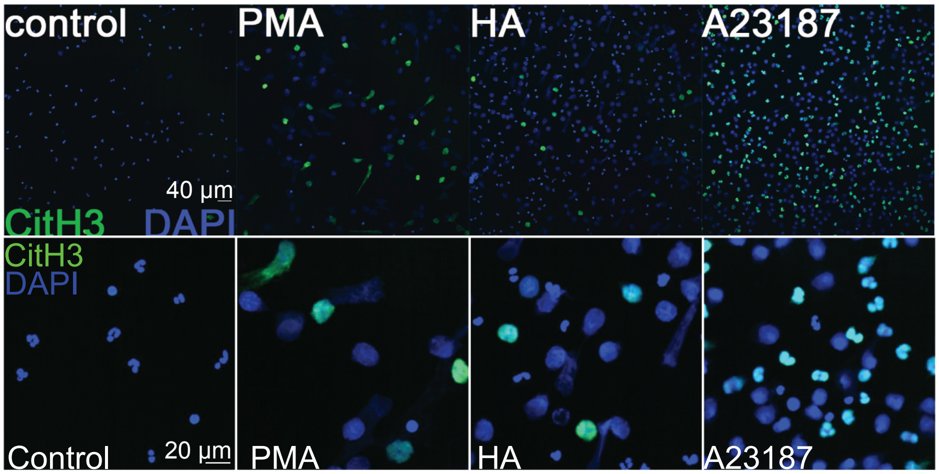

c

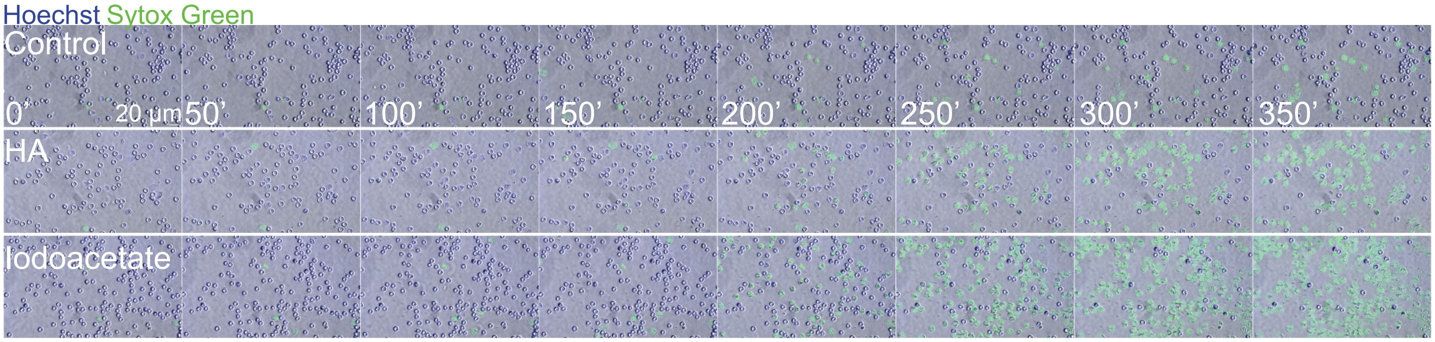

Supplementary Figure 4. GAPDH inhibition promotes NET formation
(a) Schematic of time-lapse microscopy setup
(b) Histone citrullination in primary human neutrophils after PMA or HA or A23187 treatment. First row shows all cells in field of view, second row shows magnified images.
(c) Time-lapse imaging of primary human neutrophils treated with HA (100 µM) or iodoacetate (500 µM). N = 3 independent experiments.

FIGURE S5

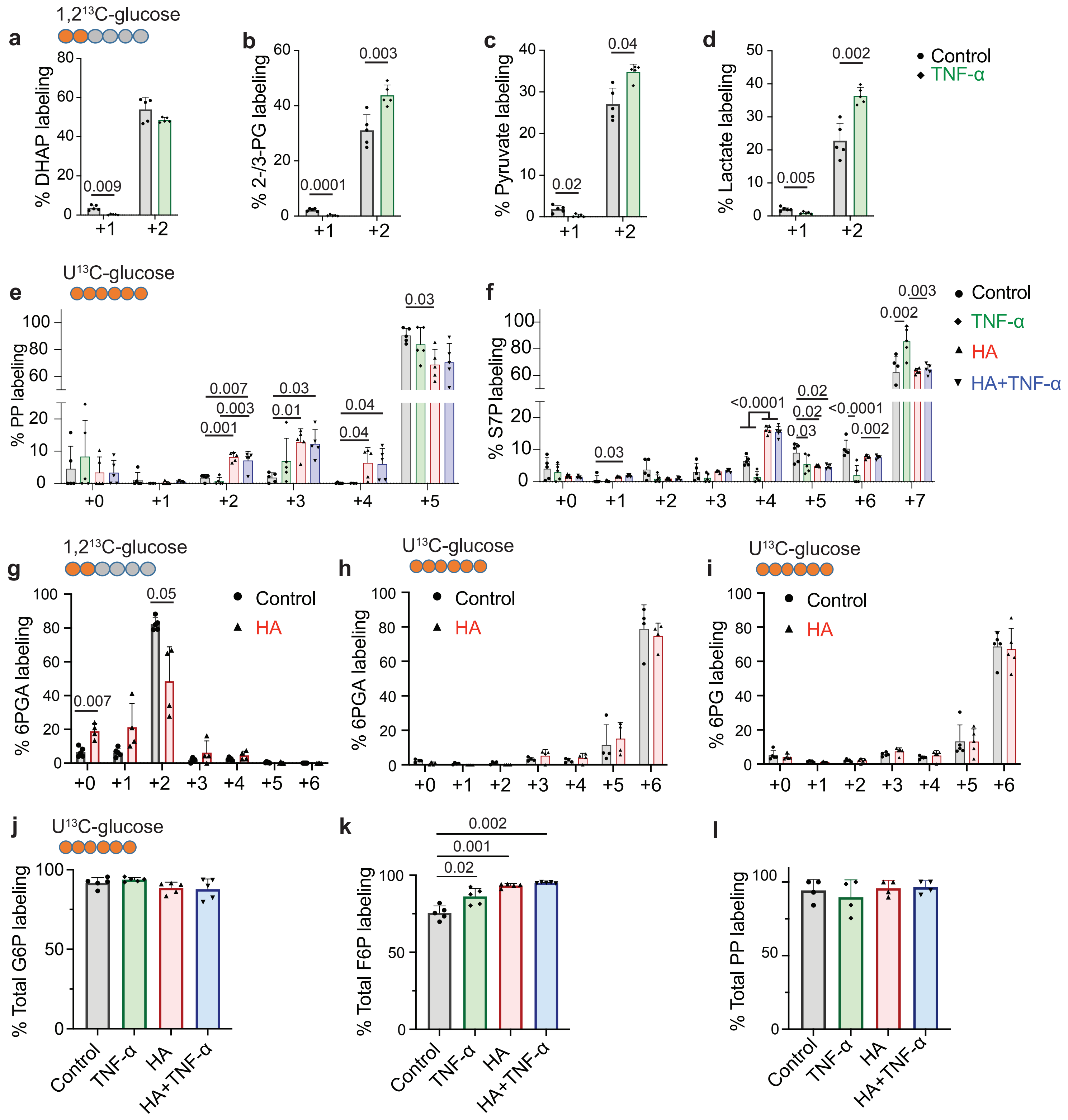

Supplementary Figure 5. Control of neutrophil glycolysis and pentose phosphate pathway activity by GAPDH and TNF-α
(a-d) Dihydroxyacetone phosphate, 3-phosphoglycerate, pyruvate, and lactate m+1 and m+2 isotopologues after 1,2^{13}C-glucose tracing.
(e-f) Pentose phosphate (PP) and sedoheptulose 7-phosphate (S7P) isotopologues after U^{13}C-glucose tracing.
(g) 6-phosphogluconolactone (6PGA) isotopologues after 1,2^{13}C-glucose tracing.
(h-i) 6-phosphogluconolactone (6PGA) and 6-phosphogluconate (6PG) isotopologues after U^{13}C-glucose tracing.
(j-l) Glucose 6-phosphate, fructose 6-phosphate, and pentose phosphate (the sum of all labeled isotopologues) after U^{13}C-glucose tracing.
Data represent mean ± st.dev. n = 5 donors from 5 independent experiments. Statistical significance was assessed with two-tailed Welch's t-test with Holm-Sidak multiple comparisons correction (a, c-m+2), two-tailed t-test with Holm-Sidak multiple comparisons correction (b, c-m+1, d, g), one-way ANOVA with Holm-Sidak multiple comparisons correction (e, f), and one-way ANOVA with Brown-Forsythe correction and Dunnett's T3 multiple comparisons test (k).

FIGURE S6

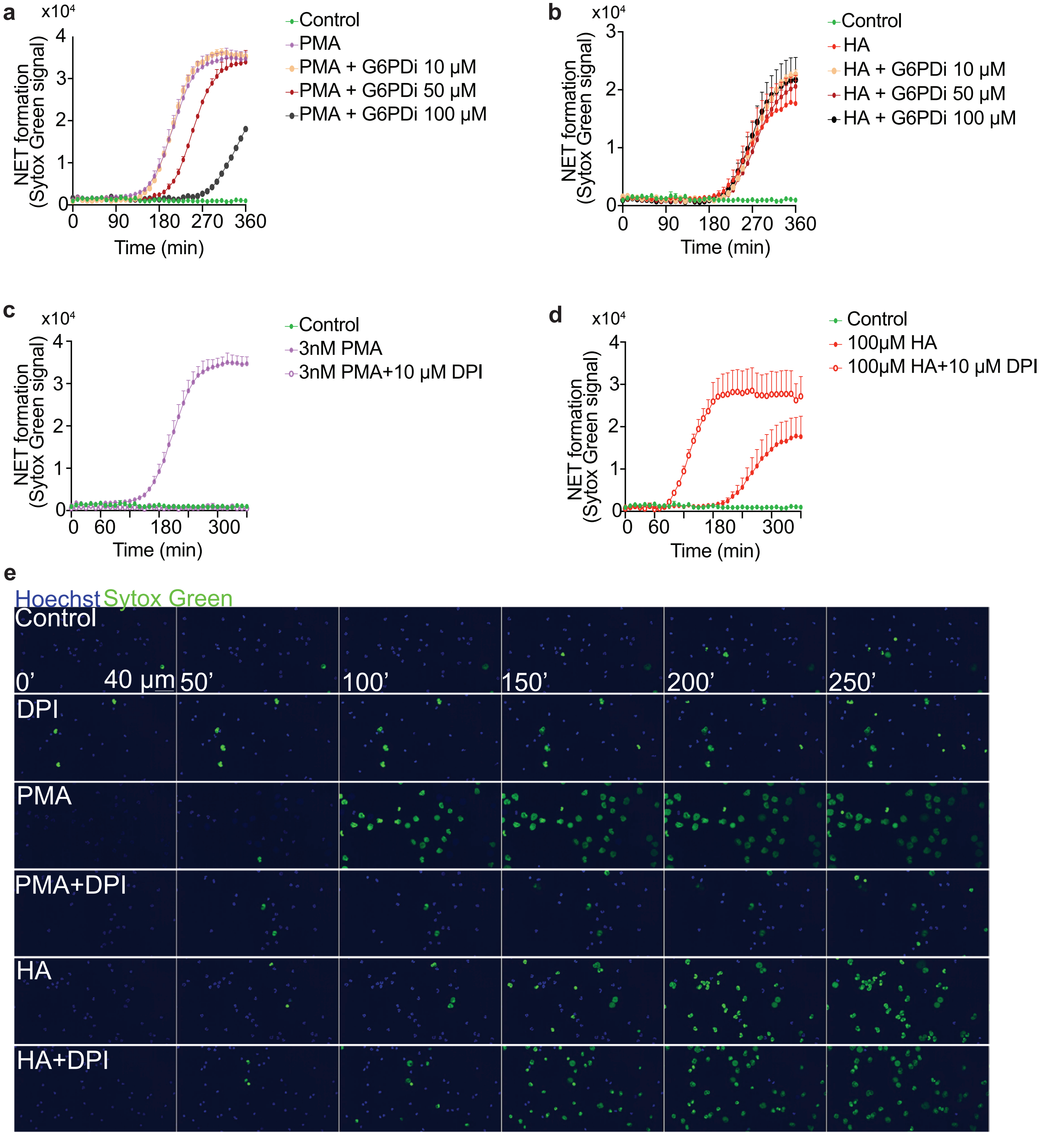

Supplementary Figure 6. Induction of NETs by GAPDH inhibition is independent of the oxPPP or NOX activity
(a, b) Representative Sytox Green microplate assay results of the effect of a G6PD inhibitor (G6PDi) on PMA-induced (a) or HA-induced NET formation (b). Quantitation shown in Figure 4i.
(c, d) Representative Sytox Green microplate assay results of the effect of NOX inhibition by DPI on PMA-induced (c) or HA-induced NET formation (d). Quantitation shown in Figure 4j.
(e) Time-lapse imaging of primary human neutrophils treated with HA (100 μM), PMA (15 nM), with or without diphenyleneiodonium (DPI) (10 μM). n = 3 independent experiments.
Data in a-d represent mean ± s.e.m. from one representative experiment of a total of 3 (a-b) or 5 (c-d) independent experiments.

FIGURE S7

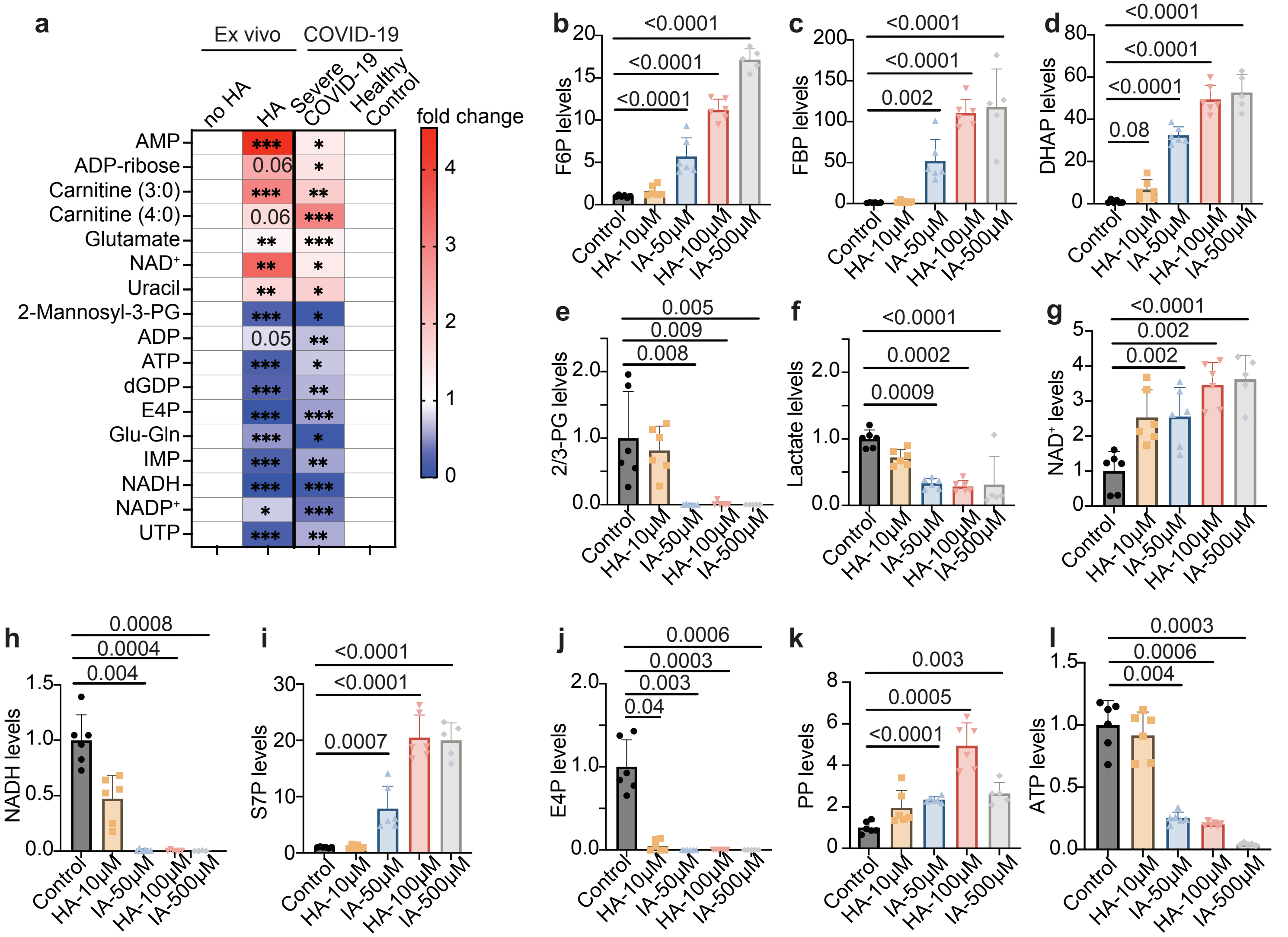

Supplementary Figure 7. Metabolic consequences of GAPDH inhibition

(a) Metabolic similarities between neutrophils treated with HA and neutrophils from severe COVID-19 patients.

(b-l) Effects of two doses of HA or iodoacetate treatment on levels of glycolytic and pentose phosphate pathway metabolites.

N = 6 samples/treatment from 3 donors in 3 independent experiments.

Data represent mean ± st.dev. In a, $^*p < 0.05$, $^{**}p < 0.01$, $^{***}p < 0.001$, and corresponding p values are provided in supplementary materials. Statistical significance was assessed with two tailed t test followed by multiple comparisons correction (a), one-way ANOVA with Holm-Sidak multiple comparisons correction (b, c, d, g, i), one-way ANOVA on log-transformed values with Holm-Sidak multiple comparisons correction (f), Kruskal-Wallis with Dunn's multiple comparisons correction (e, h, j), one-way ANOVA with Brown-Forsythe correction and Dunnett's T3 multiple comparisons test (k, l).

FIGURE S8

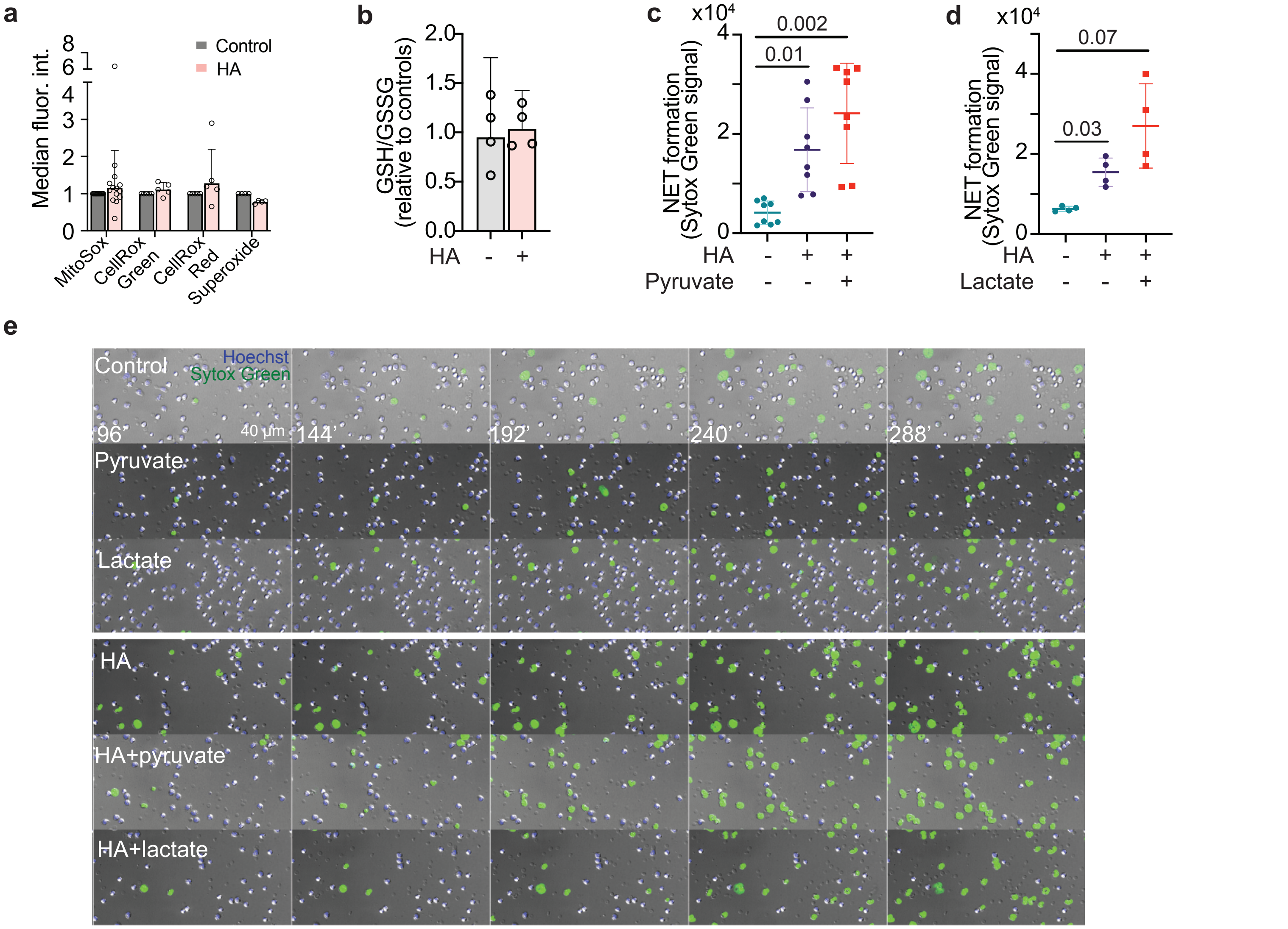

Supplementary Figure 8. Induction of NETs by GAPDH inhibition is independent of pyruvate or lactate
(a-b) Effects of HA treatment on ROS and the GSH/GSSG ratio. N = 4-14 donor samples/treatment from 4-14 independent experiments for a, and n = 4 donor samples/treatment from 4 independent experiments for b.
(c-d) Pyruvate or lactate treatment does not rescue the effects of HA treatment on Sytox Green fluorescent signal intensity in primary human neutrophils. In c, n = 8 samples/treatment from 4 donors assessed in 4 independent experiments. In d, n = 4 samples/treatment from 2 donors assessed in 2 independent experiments
(e) Time-lapse imaging of primary human neutrophils treated with HA (100 µM) with or without pyruvate (5 mM) or lactate (5 mM). Data represent mean ± st.dev (c-d) or geometric mean ± st.dev (a-b). p values are shown on the graphs. Statistical significance was assessed with one-way ANOVA with Brown-Forsythe correction and Dunnett's T3 multiple comparisons test (c, d). For some of the experiments in panels c and d, pyruvate+HA and lactate+HA treatments were tested in parallel with the same control or HA treatments.

FIGURE S9

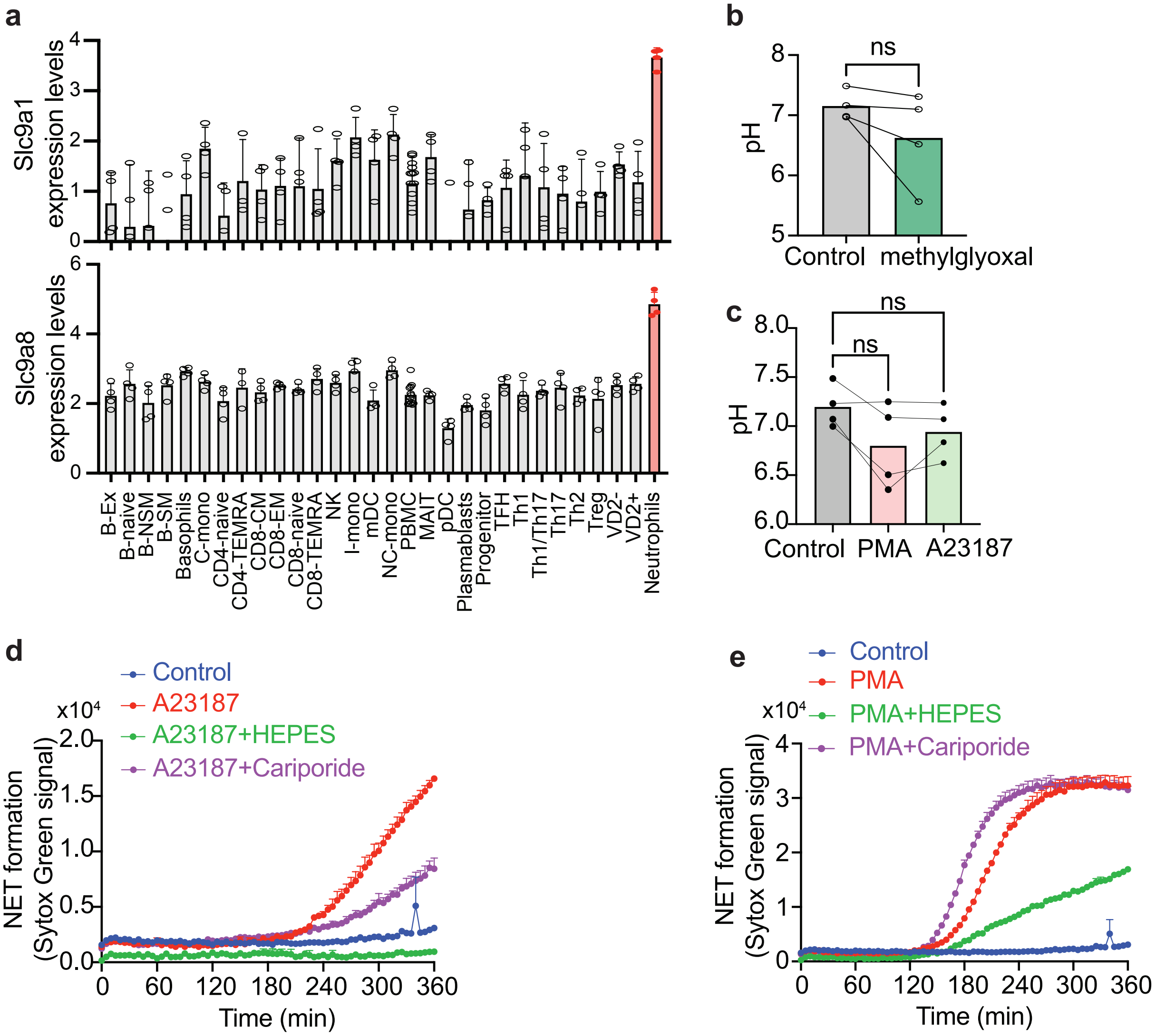

Supplementary Figure 9. Effects of PMA, A23187 and methylglyoxal on neutrophil pH
(a) Expression of Slc9a1 and Slc9a8 in neutrophils and other human blood cell types (data from Cell Rep 26:1627). Data represent mean ± st.dev.
(b) Neutrophil pH after treatment with 125 μM methylglyoxal for 1 hour. n = 4 donors from 4 independent experiments.
(c) Neutrophil pH after A23187 or PMA treatment for 30 minutes. n = 4 donors from 4 independent experiments.
(d, e) Representative Sytox Green microplate assay results of the effect of HEPES and cariporide on A23187-induced or PMA-induced NET formation. Data represent mean ± s.e.m. Representative data shown from 1 experiment of a total of n = 3 donors from 3 independent experiments.

FIGURE S10

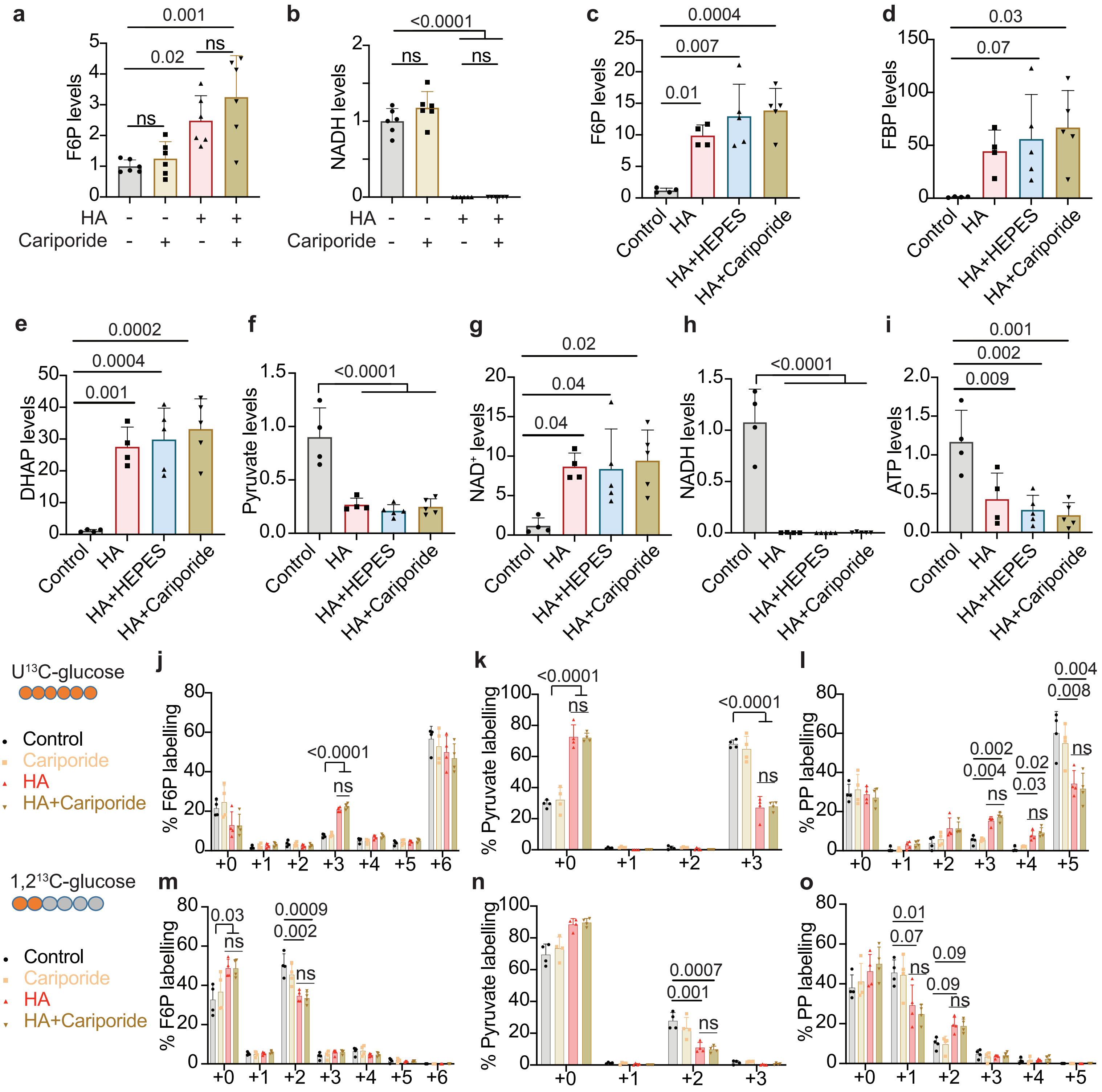

Supplementary Figure 10. Normalization of the pH does not reverse metabolic changes caused by GAPDH inhibition
(a, b) Metabolite levels, normalized to controls, in human neutrophils treated with cariporide and/or HA. n = 6 samples/treatment from 3 donors.
(c-i) Metabolite levels, normalized to controls, in human neutrophils treated with cariporide or HEPES and/or HA. n = 4-5 samples/treatment from 3 donors
(j-l) Isotopologue fractional abundance in indicated metabolites after U13C-glucose tracing in human neutrophils treated with cariporide and/or HA. n = 4 samples/treatment from 2 donors.
(m-o) Isotopologue fractional abundance in indicated metabolites after 1,213C-glucose tracing in human neutrophils treated with cariporide and/or HA. n = 4 samples/treatment from 2 donors.
Data represent mean ± st.dev. p values are shown on the graphs. Statistical significance was assessed with one-way ANOVA with Holm-Sidak multiple comparisons correction (a-k, m-n), one-way ANOVA with Brown-Forsythe correction and Dunnett's T3 multiple comparisons test (l), or Kruskal Wallis tests (o-m+2).

FIGURE S11

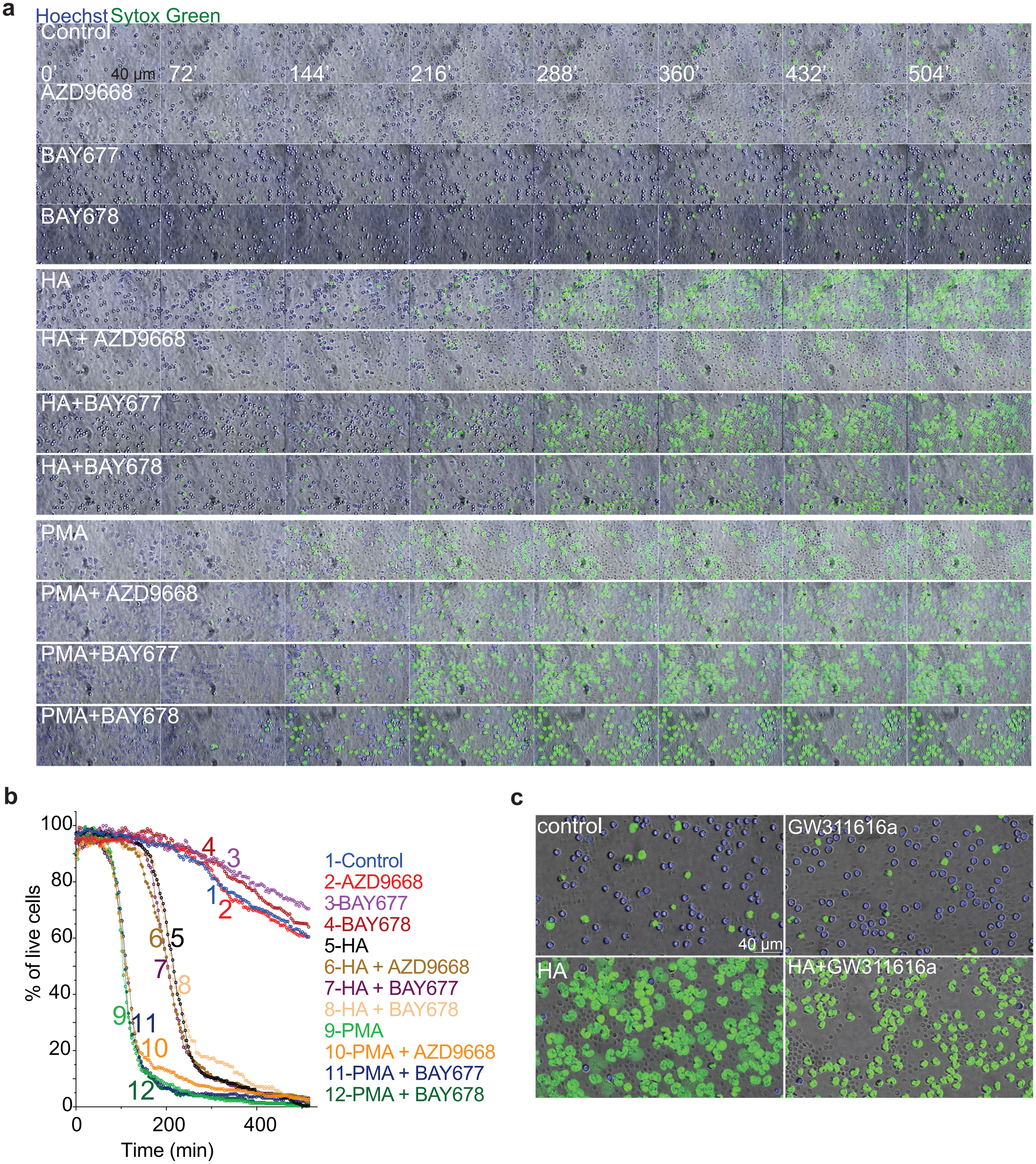

Supplementary Figure 11. GAPDH inhibition-induced NET formation depends on neutrophil elastase

(a) Time-lapse imaging of primary human neutrophils treated with HA (100 µM) or PMA (15 nM) with or without neutrophil elastase inhibitor AZD9668 (5 µM) or neutrophil elastase inhibitor BAY-678 (10 µM) or the inactive (negative control) compound BAY-677 (10 µM).

(b) Quantification of the onset of neutrophil death in the treatments shown, as assessed by the % of Sytox Green-positive nuclei. PMA treatment causes neutrophil death earlier than HA. Neutrophil elastase inhibitors do not delay neutrophil death in response to either treatment.

(c) Effect of neutrophil elastase inhibitor GW311616a (5 µM) on HA-induced NET formation. Shown is one frame/treatment from time-lapse movie.
