# Supplementary material for: Neutrophil metabolomics in severe COVID-19 reveal GAPDH as a suppressor of neutrophil extracellular trap formation
Source: Nat Commun. 2023 May 5;14:2610. doi: 10.1038/s41467-023-37567-w (PMC10162006; doi:10.1038/s41467-023-37567-w)
Supplement: Supplementary file 3 — Reporting Summary [file 41467_2023_37567_MOESM3_ESM.pdf]

nature portfolio

Corresponding author(s): Michalis Agathocleous

Last updated by author(s): 03/07/2023

Reporting Summary

Nature Portfolio wishes to improve the reproducibility of the work that we publish. This form provides structure for consistency and transparency in reporting. For further information on Nature Portfolio policies, see our Editorial Policies and the Editorial Policy Checklist.

Statistics

For all statistical analyses, confirm that the following items are present in the figure legend, table legend, main text, or Methods section.

n/a	Confirmed	
☐	☒	The exact sample size (*n*) for each experimental group/condition, given as a discrete number and unit of measurement
☐	☒	A statement on whether measurements were taken from distinct samples or whether the same sample was measured repeatedly
☐	☒	The statistical test(s) used AND whether they are one- or two-sided<br>*Only common tests should be described solely by name; describe more complex techniques in the Methods section.*
☐	☒	A description of all covariates tested
☐	☒	A description of any assumptions or corrections, such as tests of normality and adjustment for multiple comparisons
☐	☒	A full description of the statistical parameters including central tendency (e.g. means) or other basic estimates (e.g. regression coefficient) AND variation (e.g. standard deviation) or associated estimates of uncertainty (e.g. confidence intervals)
☐	☒	For null hypothesis testing, the test statistic (e.g. *F*, *t*, *r*) with confidence intervals, effect sizes, degrees of freedom and *P* value noted<br>*Give P values as exact values whenever suitable.*
☒	☐	For Bayesian analysis, information on the choice of priors and Markov chain Monte Carlo settings
☒	☐	For hierarchical and complex designs, identification of the appropriate level for tests and full reporting of outcomes
☒	☐	Estimates of effect sizes (e.g. Cohen's *d*, Pearson's *r*), indicating how they were calculated

Our web collection on statistics for biologists contains articles on many of the points above.

Software and code

Policy information about availability of computer code

Data collection	No custom code was used for data collection.
Data analysis	GraphPad Prism v9.0 & v9.3 and R were used for statistical tests. Cytoscape was used to visualize metabolite networks. Multiquant 2.1.1, MetaboAnalyst v5, Compound Discoverer 3.1 and Tracefinder 5.1 were used to analyze metabolomics data. Fiji (ImageJ2) v2.3.0 was used for image data analysis. FlowJo v10 was used to analyze flow cytometry data.

For manuscripts utilizing custom algorithms or software that are central to the research but not yet described in published literature, software must be made available to editors and reviewers. We strongly encourage code deposition in a community repository (e.g. GitHub). See the Nature Portfolio guidelines for submitting code & software for further information.

Data

Policy information about availability of data

All manuscripts must include a data availability statement. This statement should provide the following information, where applicable:

- Accession codes, unique identifiers, or web links for publicly available datasets
- A description of any restrictions on data availability
- For clinical datasets or third party data, please ensure that the statement adheres to our policy

Data availability: The metabolomics data from COVID-19 patient or healthy control neutrophils have been deposited in Metabolomics Workbench under the DOI for this project (PR001600): http://dx.doi.org/10.21228/M8W70C. Metabolomics datasets analyzed in the current study are included in the supplementary tables. Gene

expression data for neutrophils were obtained as described from Monaco et al (PMID: 30726743) or from the Bloodspot/BLUEPRINT database (PMID: 30395307)

Human research participants

Policy information about studies involving human research participants and Sex and Gender in Research.

Reporting on sex and gender	Patient gender was determined according to standard clinical practice. Gender was considered as a variable in statistical adjustment for confounders. Separate analysis in male and female patients was not performed because of limited number of available samples.
Population characteristics	Age, gender, and some clinical characteristics are shown in Figure S1. Adjustments for these confounders was performed as described in methods.
Recruitment	COVID-19 patient samples were obtained through the University of Texas Southwestern Medical Center biorepository. Sample collection from COVID-19 patients or healthy controls was performed according to procedures approved by the Institutional Review Board of the University of Texas Southwestern Medical Center (STU-2020-0375 for SARS-CoV2 Biorepository and STU 012014-040 to J. Moreland) and the UT Southwestern Biorepository.
Ethics oversight	Institutional Review Board of the University of Texas Southwestern Medical Center

Note that full information on the approval of the study protocol must also be provided in the manuscript.

Field-specific reporting

Please select the one below that is the best fit for your research. If you are not sure, read the appropriate sections before making your selection.

☒ Life sciences ☐ Behavioural & social sciences ☐ Ecological, evolutionary & environmental sciences

For a reference copy of the document with all sections, see nature.com/documents/nr-reporting-summary-flat.pdf

Life sciences study design

All studies must disclose on these points even when the disclosure is negative.

Sample size	Sample size: No statistical methods were used to predetermine sample size. Sample size for patient metabolomics analysis was determined by sample availability at the time of analysis. For experiments other than patient metabolomics analysis sample size was partly limited by donor availability. n=3-10 independent experiments were typically performed as specified in figure legends.
Data exclusions	No data were excluded from the analysis
Replication	For experimental results in which graphs are shown, aggregate results from all independent experiments are presented. For experimental results in which representative images are shown, e.g. microscopy results, all performed experiments showed the reported findings. The number of independent replications for each experiment is indicated in figure legends.
Randomization	For patient samples, UT Southwestern Biorepository samples available at the time of analysis which fulfilled pre-specified clinical criteria were used. Patients in the ‘mild’ category are defined as ‘mild or moderate’ and patients in the ‘severe’ category are defined as ‘critical’ by WHO severity definitions (https://www.who.int/publications/i/item/WHO-2019-nCoV-clinical-2021-2). For mass spec analysis, samples were randomized to avoid effects of systematic drift. For experiments using neutrophils from healthy donors, matched control and treatment conditions were used for each donor sample. Samples were randomized for mass spec analysis.
Blinding	Experimenters were not blinded to group allocation. Samples from all treatment groups were analyzed in parallel in experiments.

Reporting for specific materials, systems and methods

We require information from authors about some types of materials, experimental systems and methods used in many studies. Here, indicate whether each material, system or method listed is relevant to your study. If you are not sure if a list item applies to your research, read the appropriate section before selecting a response.

Materials & experimental systems

n/a	Involved in the study	
☐	☒	Antibodies
☒	☐	Eukaryotic cell lines
☒	☐	Palaeontology and archaeology
☒	☐	Animals and other organisms
☒	☐	Clinical data
☒	☐	Dual use research of concern

Methods

n/a	Involved in the study	
☒	☐	ChIP-seq
☐	☒	Flow cytometry
☒	☐	MRI-based neuroimaging

Antibodies

Antibodies used	anti-human CD49d-APC (clone 9F10, Biolegend cat number 304308), anti-human CD15-577 FITC (clone HI98, Biolegend cat number 301904), rabbit anti-neutrophil elastase (Sigma, #481001), rabbit anti-citrullinated histone H3 (Abcam, # ab5103) and Alexa Fluor 488-Affinipure goat anti-rabbit IgG (Jackson Immuno Research, Fisher #NC0323535)
Validation	These antibodies are commonly used for neutrophil identification and studies of NET formation (e.g. PMID: 25799053, 33936029, 35522219, 35852866, 31628160, 28574339, 31775038, 34320407)

Flow Cytometry

Plots

Confirm that:

☒ The axis labels state the marker and fluorochrome used (e.g. CD4-FITC).

☒ The axis scales are clearly visible. Include numbers along axes only for bottom left plot of group (a 'group' is an analysis of identical markers).

☒ All plots are contour plots with outliers or pseudocolor plots.

☒ A numerical value for number of cells or percentage (with statistics) is provided.

Methodology

Sample preparation	Primary human polymorphonuclear leukocytes were isolated and stained using fluorescent antibodies or dyes as described in the methods section.
Instrument	FACSCanto and FACSAria (BD)
Software	FACSDiva (BD) and FlowJo (FlowJo LLC)
Cell population abundance	No sort was performed
Gating strategy	As shown in Figure S1. FSC/SSC, FSC-A/FSC-H (singlet), Live/dead (DAPI) gating.

☒ Tick this box to confirm that a figure exemplifying the gating strategy is provided in the Supplementary Information.
