# Supplementary material for: Neutrophil metabolomics in severe COVID-19 reveal GAPDH as a suppressor of neutrophil extracellular trap formation
Source: Nat Commun. 2023 May 5;14:2610. doi: 10.1038/s41467-023-37567-w (PMC10162006; doi:10.1038/s41467-023-37567-w)
Supplement: Supplementary file 4 — Description of Additional Supplementary Files [file 41467_2023_37567_MOESM4_ESM.docx]

**Description of additional supplementary files**

Supplementary Data 1

Description: Patient metabolomic data analysis.

Supplementary Data 2

Description: Metabolites whose levels significantly change in healthy control vs mild vs severe COVID-19 neutrophils.

Supplementary Data 3

Description: Patient metabolomic data analysis after adjustment for age, gender and sample collection time variables.

Supplementary Data 4

Description: Metabolites whose levels significantly change in healthy control vs mild vs severe COVID-19 neutrophils after adjustment for age, gender and sample collection time variables.

Supplementary Data 5

Description: p values and relative changes for metabolites whose levels significantly change in both control vs HA treated neutrophils and healthy control vs severe COVID-19 patient neutrophil comparisons (related to Figure S7a).

Supplementary Movie 1: movie001_control

Description: Time-lapse of neutrophils in control treatment corresponding to Fig. 2e-f

Supplementary Movie 2: movie002_HA

Description: Time-lapse of neutrophils in HA treatment corresponding to Fig. 2e-f

Supplementary Movie 3: movie003_PMA

Description: Time-lapse of neutrophils in PMA treatment corresponding to Fig. 2e-f

Supplementary Movie 4: movie004_control

Description: Time-lapse of neutrophils in control treatment corresponding to Fig. 2g

Supplementary Movie 5: movie005_HA

Description: Time-lapse of neutrophils in HA treatment corresponding to Fig. 2g

Supplementary Movie 6: movie006_PMA

Description: Time-lapse of neutrophils in PMA treatment corresponding to Fig. 2g

Supplementary Movie 7: movie007_A23187

Description: Time-lapse of neutrophils in A23187 treatment corresponding to Fig. 2g

Supplementary Movie 8: movie008_control

Description: Time-lapse of neutrophils in control treatment corresponding to Fig. S4c

Supplementary Movie 9: movie009_HA

Description: Time-lapse of neutrophils in HA treatment corresponding to Fig. S4c

Supplementary Movie 10: movie010_IA

Description: Time-lapse of neutrophils in iodoacetate treatment corresponding to Fig. S4c

Supplementary Movie 11: movie011_control

Description: Time-lapse of neutrophils in control treatment corresponding to Fig. 4k, S6e

Supplementary Movie 12: movie012_DPI

Description: Time-lapse of neutrophils in DPI treatment corresponding to Fig. 4k, S6e

Supplementary Movie 13: movie013_PMA

Description: Time-lapse of neutrophils in PMA treatment corresponding to Fig. 4k, S6e

Supplementary Movie 14: movie014_PMA+DPI

Description: Time-lapse of neutrophils in PMA + DPI treatment corresponding to Fig. 4k, S6e

Supplementary Movie 15: movie015_HA

Description: Time-lapse of neutrophils in HA treatment corresponding to Fig. 4k, S6e

Supplementary Movie 16: movie016_HA+DPI

Description: Time-lapse of neutrophils in HA + DPI treatment corresponding to Fig. 4k, S6e

Supplementary Movie 17: movie017_control

Description: Time-lapse of neutrophils in control treatment corresponding to Fig. S8e

Supplementary Movie 18: movie018_pyruvate

Description: Time-lapse of neutrophils in pyruvate treatment corresponding to Fig. S8e

Supplementary Movie 19: movie019_lactate

Description: Time-lapse of neutrophils in lactate treatment corresponding to Fig. S8e

Supplementary Movie 20: movie020_HA

Description: Time-lapse of neutrophils in HA treatment corresponding to Fig. S8e

Supplementary Movie 21: movie021_HA+pyruvate

Description: Time-lapse of neutrophils in HA + pyruvate treatment corresponding to Fig. S8e

Supplementary Movie 22: movie022_ HA+lactate

Description: Time-lapse of neutrophils in HA + lactate treatment corresponding to Fig. S8e

Supplementary Movie 23: movie023_control

Description: Time-lapse of neutrophils in control treatment corresponding to Fig. 5j

Supplementary Movie 24: movie024_2DG

Description: Time-lapse of neutrophils in 2DG treatment corresponding to Fig. 5j

Supplementary Movie 25: movie025_HA

Description: Time-lapse of neutrophils in HA treatment corresponding to Fig. 5j

Supplementary Movie 26: movie026_HA+2DG

Description: Time-lapse of neutrophils in HA + 2DG treatment corresponding to Fig. 5j

Supplementary Movie 27: movie027_control

Description: Time-lapse of neutrophils in control treatment corresponding to Fig. 6c

Supplementary Movie 28: movie028_HA

Description: Time-lapse of neutrophils in HA treatment corresponding to Fig. 6c

Supplementary Movie 29: movie029_HEPES

Description: Time-lapse of neutrophils in HEPES treatment corresponding to Fig. 6c

Supplementary Movie 30: movie030_HA+HEPES

Description: Time-lapse of neutrophils in HA + HEPES treatment corresponding to Fig. 6c

Supplementary Movie 31: movie031_cariporide

Description: Time-lapse of neutrophils in cariporide treatment corresponding to Fig. 6c

Supplementary Movie 32: movie032_HA+cariporide

Description: Time-lapse of neutrophils in HA + cariporide treatment corresponding to Fig. 6c

Supplementary Movie 33: movie033_control

Description: Time-lapse of neutrophils in control treatment corresponding to Fig. 6j, S11a

Supplementary Movie 34: movie034_AZD9668

Description: Time-lapse of neutrophils in AZD9668 treatment corresponding to Fig. 6j, S11a

Supplementary Movie 35: movie035_ BAY677

Description: Time-lapse of neutrophils in BAY-677 treatment corresponding to Fig. 6j, S11a

Supplementary Movie 36: movie036_ BAY678

Description: Time-lapse of neutrophils in BAY-678 treatment corresponding to Fig. 6j, S11a

Supplementary Movie 37: movie037_HA

Description: Time-lapse of neutrophils in HA treatment corresponding to Fig. 6j, S11a

Supplementary Movie 38: movie038_HA+AZD9668

Description: Time-lapse of neutrophils in HA + AZD9668 treatment corresponding to Fig. 6j, S11a

Supplementary Movie 39: movie039_HA+BAY677

Description: Time-lapse of neutrophils in HA + BAY-677 treatment corresponding to Fig. 6j, S11a

Supplementary Movie 40: movie040_ HA+BAY678

Description: Time-lapse of neutrophils in HA + BAY-678 treatment corresponding to Fig. 6j, S11a

Supplementary Movie 41: movie041_PMA

Description: Time-lapse of neutrophils in PMA treatment corresponding to Fig. 6j, S11a

Supplementary Movie 42: movie042_PMA+AZD9668

Description: Time-lapse of neutrophils in PMA + AZD9668 treatment corresponding to Fig. 6j, S11a

Supplementary Movie 43: movie043_PMA+BAY677

Description: Time-lapse of neutrophils in PMA + BAY-677 treatment corresponding to Fig. 6j, S11a

Supplementary Movie 44: movie044_PMA+BAY678

Description: Time-lapse of neutrophils in PMA + BAY-678 treatment corresponding to Fig. 6j, S11a
